# A macrophage related signature for predicting prognosis and drug sensitivity in ovarian cancer based on integrative machine learning

**DOI:** 10.1186/s12920-023-01671-z

**Published:** 2023-10-02

**Authors:** Bo Zhao, Lipeng Pei

**Affiliations:** Department of Obstetrics and Gynecology, General Hospital of Northern Theater Command, Shenyang, 110016 China

**Keywords:** Macrophage, Machine learning, Ovarian cancer, Prognostic signature, Immunotherapy

## Abstract

**Background:**

Ovarian cancer ranks the leading cause of gynecologic cancer-related death in the United States and the fifth most common cause of cancer-related mortality among American women. Increasing evidences have highlighted the vital role of macrophages M2/M1 proportion in tumor progression, prognosis and immunotherapy.

**Methods:**

Weighted gene co-expression network analysis (WGCNA) was performed to identify macrophages related markers. Integrative procedure including 10 machine learning algorithms were performed to develop a prognostic macrophage related signature (MRS) with TCGA, GSE14764, GSE140082 datasets. The role of MRS in tumor microenvironment (TME) and therapy response was evaluated with the data of CIBERSORT, MCPcounter, QUANTISEQ, XCELL, CIBERSORT-ABS, TIMER and EPIC, GSE91061 and IMvigor210 dataset.

**Results:**

The optimal MRS developed by the combination of CoxBoost and StepCox[forward] algorithm served as an independent risk factor in ovarian cancer. Compared with stage, grade and other established prognostic signatures, the current MRS had a better performance in predicting the overall survival rate of ovarian cancer patients. Low risk score indicated a higher TME score, higher level of immune cells, higher immunophenoscore, higher tumor mutational burden, lower TIDE score and lower IC50 value in ovarian cancer. The survival prediction nomogram had a good potential for clinical application in predicting the 1-, 3-, and 5-year overall survival rate of ovarian cancer patients.

**Conclusion:**

All in all, the current study constructed a powerful prognostic MRS for ovarian cancer patients using 10 machine learning algorithms. This MRS could predict the prognosis and drug sensitivity in ovarian cancer.

**Supplementary Information:**

The online version contains supplementary material available at 10.1186/s12920-023-01671-z.

## Introduction

Ovarian cancer ranks the leading cause of gynecologic cancer-related death in the United States and the fifth most common cause of cancer-related mortality among American women [1]. Each year, a total of 140 000 women are estimated to die from ovarian cancer globally [2]. Most of patients are already at an advanced stage when initially being diagnosed with ovarian cancer on account of limited effective screening approaches and clinical symptoms [3]. Worse still, more than half of ovarian cancer patients will suffer from relapse after standard of care therapies and the 5-year survival rate are only about 30% [4]. Tumor recurrence and metastasis and drug resistance are the main causes of the treatment failure of ovarian cancer [5]. There are limited effective biomarkers for predicting the prognosis and drug sensitivity of ovarian cancer clinically apart from FIGO staging system.

The cross-talk between ovarian cancer and tumor immune microenvironment is crucial for ovarian cancer progression and metastasis and even drug resistance [6]. Tumor-associated macrophages (TAMs) constitute the most essential immune cells present in the ovarian tumor immune microenvironment [7]. TAMs could be categorized into two functionally contrasting subtypes, namely classical activated macrophages M1 and replacement of activated macrophages M2 [8]. Increasing evidences have highlighted the vital role of macrophages M2/M1 proportion in tumor progression, prognosis and immunotherapy [9–11]. M1 macrophages are historically regarded as anti-tumor and inhibit tumor growth by mediating immune responses [12]. However, M2-polarized macrophages are referred as pro-tumor, resulting in immune suppression and tumor angiogenesis [13, 14]. Targeting to macrophages is suggested as one of the most promising approaches for cancer therapy, including ovarian cancer [7, 15, 16]. Thus, elucidating TAMs-related markers and developing macrophages-related prognostic signature may help us monitor the prognosis and immunotherapy response of ovarian cancer.

In the current study, weighted gene co-expression network analysis (WGCNA) was performed to identify macrophages related markers based on the data obtained from cell-type Identification by Estimating Relative Subsets of RNA Transcripts x (CIBERSORTx) in TCGA ovarian cancer dataset. Based on these macrophages related markers, we developed a prognostic macrophages-related signature using10 machine learning algorithms with 3 independent public datasets. The data of our study may provide more evidences about the significant functions of macrophages in the prognosis and therapy of ovarian cancer.

## Materials and methods

### Datasets acquisition and processing

**Fig. 1:**
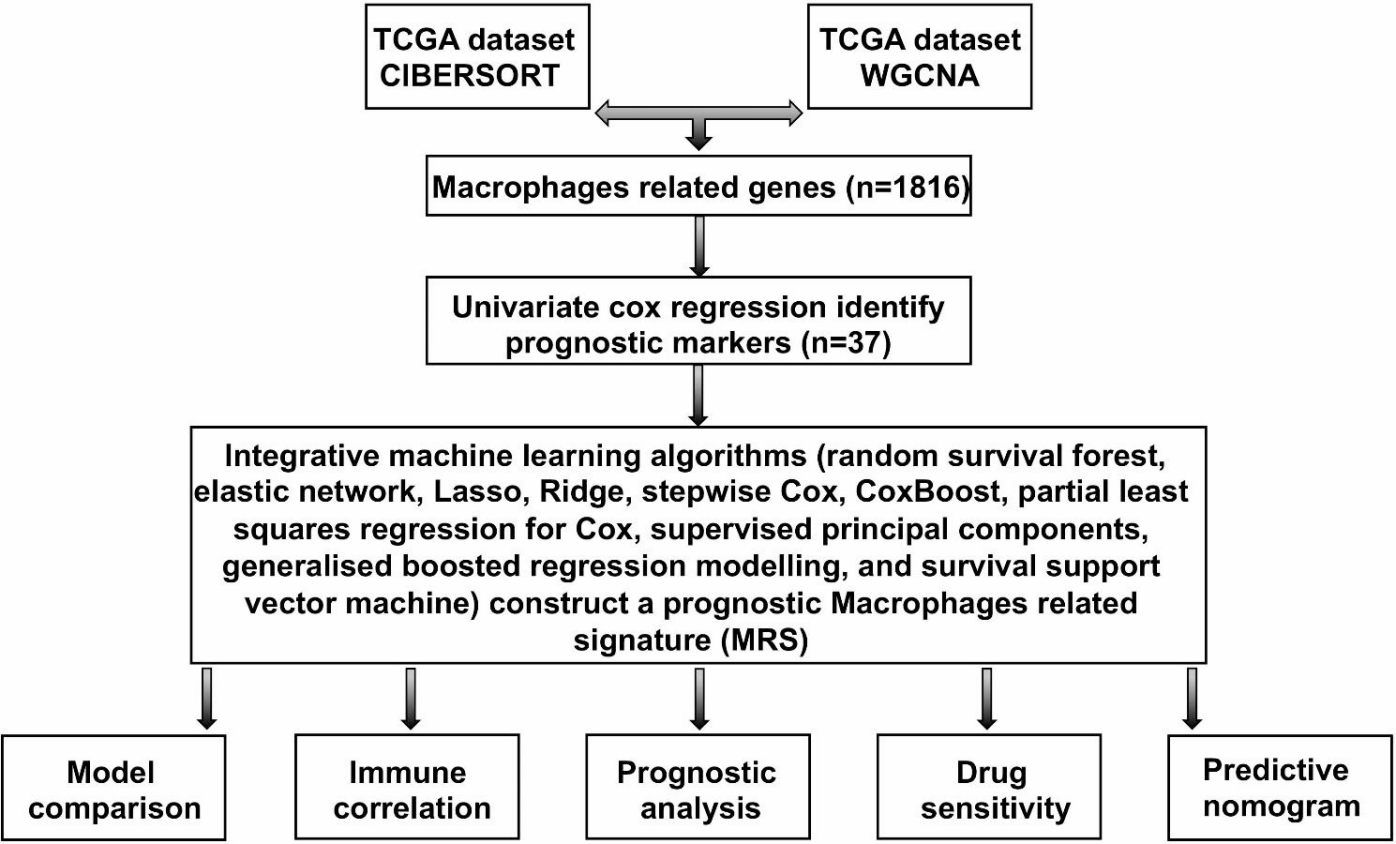
Workflow of the current study

Flow chart of the current study was shown in Fig. 1. Level 3 RNA-seq data and genomic mutation data of ovarian cancer (n = 375) were acquired from TCGA database. In order to verify the prognostic signature, another two GEO datasets (GSE14764, n = 80 and GSE140082, n = 380) were obtained. The normalization of TCGA and GEO datasets depended on R package “sva”. Two immunotherapy cohorts, including IMvigor210 (anti-PD-L1) and GSE91061 (anti-PD-L1 and anti-CTLA4), were applied to evaluate the predictive value of prognostic signature in immunotherapy.

### CIBERSORTx and WGCNA

To obtain profiling tumor infiltrating immune cells, RNA-seq data of TCGA ovarian cancer dataset was summitted to CIBERSORTx algorithm, which could calculate the expression of immune cells [17]. CIBERSORTx is a suite of machine learning tools for the assessment of cellular abundance and cell type-specific gene expression patterns from bulk tissue transcriptome profiles. In our study, the level of immune cells in each TCGA ovarian cancer case was evaluated using CIBESORTx [18]. WGCNA is a reliable tool to identify gene sets of interest from thousands of the most varied genes and clarify correlation analysis with phenotypes [19]. In this analysis, β value under the degree of independence was set as 0.9 in network construction, which could disregard weak correlations between genes in the adjacency matrix. To cluster the most representative genes, module eigengenes (MEs), the Pearson’s correlation coefficient was set as 0.25. The logic of the WGCNA method compared to other methods is that genes with high expression correlation are likely to be involved in the same biological processes or pathways and therefore can be grouped into the same modules [20]. Those genes in the module that displayed the significant positive correlation (Cor > 0.3, p < 0.001) with macrophages (M2 and M1) was defined as macrophages-related genes.

### Integrative machine learning algorithms constructed prognostic macrophages-related signature (MRS)

The potential prognostic biomarkers among macrophages-related genes in ovarian cancer were identified using univariate cox regression analysis. To construct an accurate and stable prognostic MRS in ovarian cancer, these potential prognostic biomarkers were submitted to the 10 integrative machine learning algorithms, including random survival forest (RSF), elastic network (Enet), Lasso, Ridge, stepwise Cox, CoxBoost, partial least squares regression for Cox (plsRcox), supervised principal components (SuperPC), generalized boosted regression modelling (GBM), and survival support vector machine (survival-SVM). The signature generation procedure was as follows: (1) Prognostic biomarkers were generated using Univariate Cox regression in the TCGA dataset; (2) Then, 101 algorithm combinations were performed on the prognostic signature to fit prediction models based on the leave-one-out cross-validation (LOOCV) framework in the TCGA dataset; (3) All models were detected in two GEO cohorts (GSE14764 & GSE140082); (4) For each model, the Harrell’s concordance index (C-index) was calculated across all TCGA and GEO datasets, and the model with the highest average C-index was considered optimal. Similar machine learning algorithms could be seen in previous studies [21–23]. The parameter tuning details about the R scripts in our study is available on the Github website (https://github.com/Zaoqu-Liu/IRLS).

### Evaluation of the performance of MRS

The ovarian cancer cases were divided into high and low risk group with the medium value of risk score as the cutoff. The overall survival curves were drawn with “survival” package. Moreover, Time ROC curve and clinical ROC curve generated by “timeROC” package were used to evaluate the predictive accuracy of MRS in ovarian cancer. We then randomly collected 55 prognostic models (mRNA and lncRNA-related models) that had been developed for ovarian cancer and calculated their C-indexes by using “CompareC” package.

### Correlation between risk score and immune microenvironment and genetic mutation

Immune microenvironment score of each ovarian cancer case was calculated with the ESTIMATE algorithm. The relative proportions of infiltrating immune cells were tested by immunedeconv, which could provide uniform and user-friendly access to seven state-of-the-art computational methods (CIBERSORT, MCPcounter, QUANTISEQ, XCELL, CIBERSORT-ABS, TIMER and EPIC) for deconvolution of cell-type fractions from bulk RNA-seq data [24]. The biological functions associated with Kyoto Encyclopedia of Genes and Genomes pathways in high and low risk group were explored with GSEA. The waterfall plot of SNV was generated with “maftools” package.

### Correlation between risk score and drug sensitivity

The immunophenoscore (IPS) of ovarian cancer cases were downloaded from The Cancer Immunome Atlas (TCIA, https://tcia.at/home). TIDE score and T cells dysfunction and exclusion scores of ovarian cancer cases were downloaded from TIDE (http://tide.dfci.harvard.edu), which could provide the detail of the immunotherapy response of TCGA ovarian cancer cases. Using Genomics of Drug Sensitivity in Cancer (GDSC) (https://www.cancerrxgene.org/), we generated the data of drug sensitivity. The half maximal inhibitory concentration (IC50) value of common chemotherapy and targeted drugs based on GDSC were calculated using “oncoPredict” package. In IMvigor210 and GSE91061 cohort, immunotherapy response could be divided into two parts, including responders (partial response (PR) and complete response (CR)) and non-responders (progressive disease (PD), stable disease (SD)).

### Construction of MRS based prediction nomogram

The prognostic risk factors in ovarian cancer among clinical characters and MRS were identified with univariate and multivariate cox analysis. We then construct the nomogram by utilizing R package “rms”, “nomogramEx” and “regplot” based on MRS and clinical characters for ovarian cancer.

### Validation of the expression and prognostic value of candidate markers

Immunohistochemistry about the protein level of some candidate markers were obtained from the Human Protein Atlas database (https://www.proteinatlas.org/) [25]. The Kaplan-Meier survival curve of candidate markers were generated from Kaplan-Meier Plotter (https://kmplot.com/) [26].

### Statistical analysis

R software (version 4.2.1) was used to perform statistical analyses. Wilcoxon rank-sum test or student T test was performed to compare continuous variables. The correlations between two continuous variables were evaluated with Pearson’s or Spearman’s rank correlation analysis. The two-sided log-rank test was used to test the difference in different Kaplan-Meier survival curve.

## Results

### Identification of macrophage-related genes by WGCNA in ovarian cancer

**Fig. 2:**
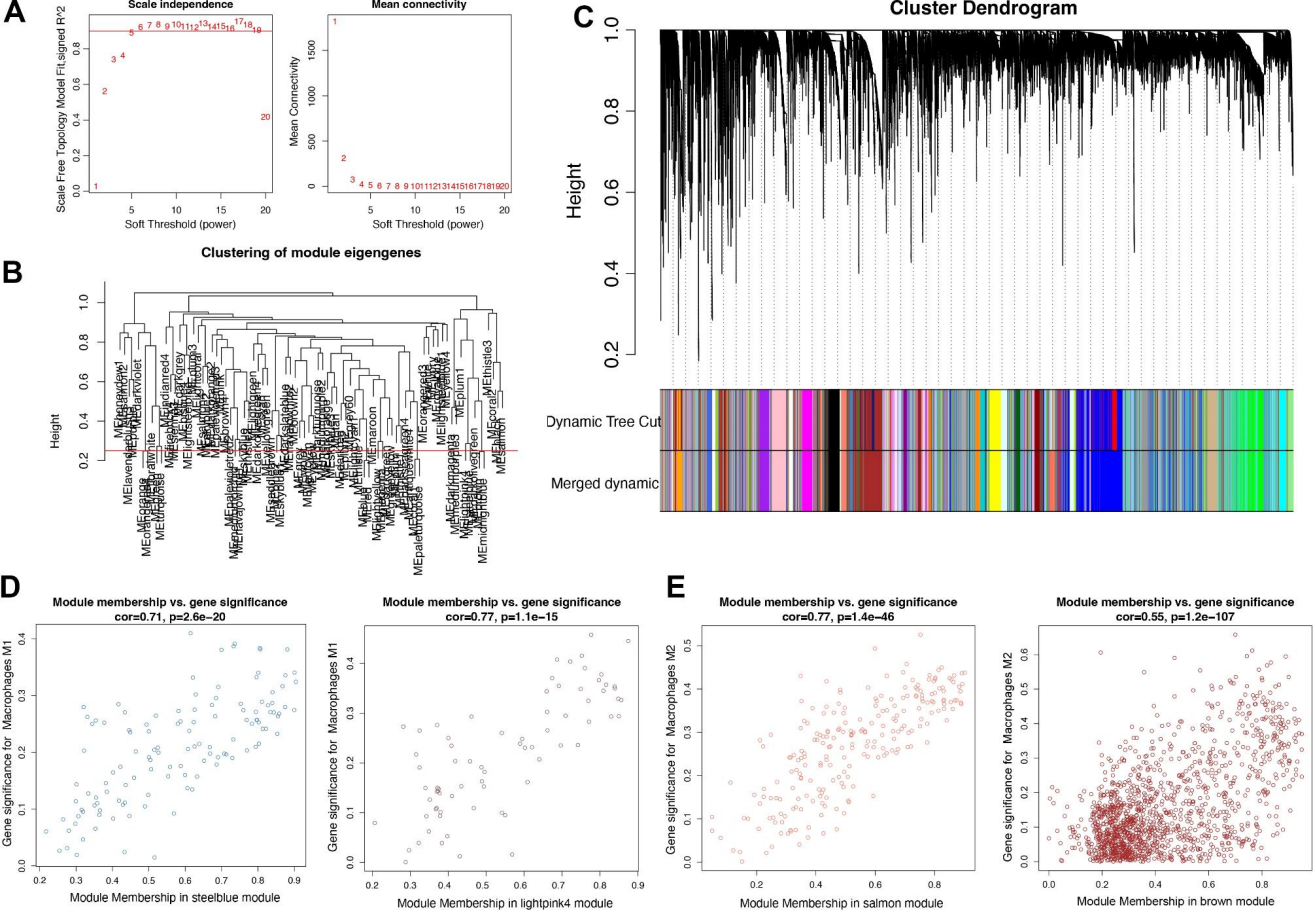
**Identification of macrophage-related markers in ovarian cancer.** (**A**) Selection of best soft threshold power. (**B**-**C**) The clustering tree of 80 differentially colored modules. (**D**-**E**) The correlation coefficient between gene significance and module membership in steelblue, lightpink4, salmon and brown module

In order to select the specific modules that were significantly with immune cells, we investigated gene expression pattern from TCGA dataset and immune cell file from CIBERSORTx algorithm. After setting β value under the degree of independence as 0.9, we obtained the optimal soft-threshold power of 5 (Fig. 2A). Using 0.25 as the cutoff, we obtained 80 modules (Fig. 2B). Figure 2 C showed the clustering tree of these 80 differentially colored modules. The correlation between modules and macrophage and other immune cells was showed in Supplementary Fig. 1. Those modules with correlation coefficient > 0.3 and P value < 0.001 were defined as immune cell related module. Based on the obtained results, it can be said that the blue module with the appropriate correlation and p-value was considered as the selected module. In this case, lightpink4 (correlation = 0.43, P = 3e-18) and steelblue (correlation = 0.35, P = 1e-12) modules were macrophage M1 related module. Macrophage M2 related modules were brown (correlation = 0.34, P = 2e-11) and salmon (correlation = 0.43, P = 1e-18) modules. The correlation coefficient between gene significance (GS) and module membership (MM) reached 0.71 and 0.77 in steelblue and lightpink4 module (Fig. 2D). The correlation coefficient between GS and MM was 0.77 and 0.55 in brown and salmon module (Fig. 2E). A total of 1816 macrophage-related genes were obtained (Supplementary Table 1).

### Integrative machine learning algorithms constructed a prognostic MRS

**Fig. 3:**
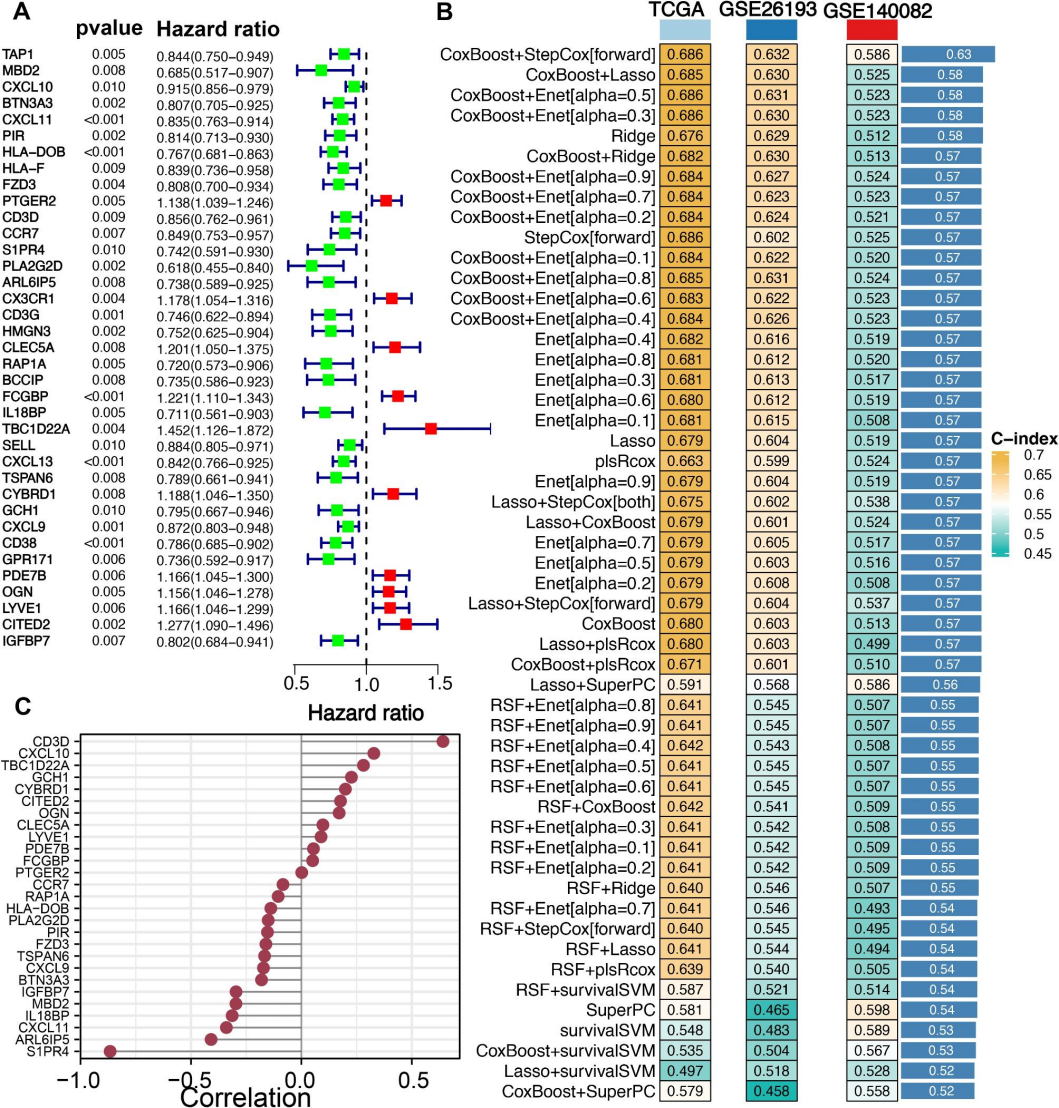
**Integrative machine learning identified a prognostic macrophages-related signature (MRS).** (**A**) Potential prognostic biomarkers identified with univariate cox analysis. (**B**) The C-index of each prognostic model constructed by 10 machine learning algorithms in training and testing cohort. (**C**) The regression coefficients of 27 genes of MRSs

As shown in Fig. 3A, a total of 37 potential prognostic macrophage-related biomarkers based on univariate cox analysis. We then submitted these 37 prognostic biomarkers into machine learning-based integrative procedure to develop an accurate and stable prognostic MRS. The C-index of each model in TCGA, GSE26193 and GSE140082 cohort was shown in Fig. 3B. Finally, the model constructed by the combination of CoxBoost and StepCox[forward] was suggested as the optimal model with the highest average C-index of 0.63 (Fig. 3B). As a result, a total of 27 macrophage-related genes was finally included in the model and this prognostic model was constructed by the combination of CoxBoost and StepCox[forward]. This consensus MRS was constructed with a final set of 27 macrophage-related biomarkers and the coefficients of each candidate gene was shown Fig. 3C and Supplementary Table 2. Based on the coefficients of 27 macrophage-related biomarkers and their expression pattern, we then calculated the risk score for each ovarian cancer patient. Ovarian cancer patients were divided into high and low risk groups with the medium value of risk score as the cutoff.

### Evaluation of the performance of MRS

**Fig. 4:**
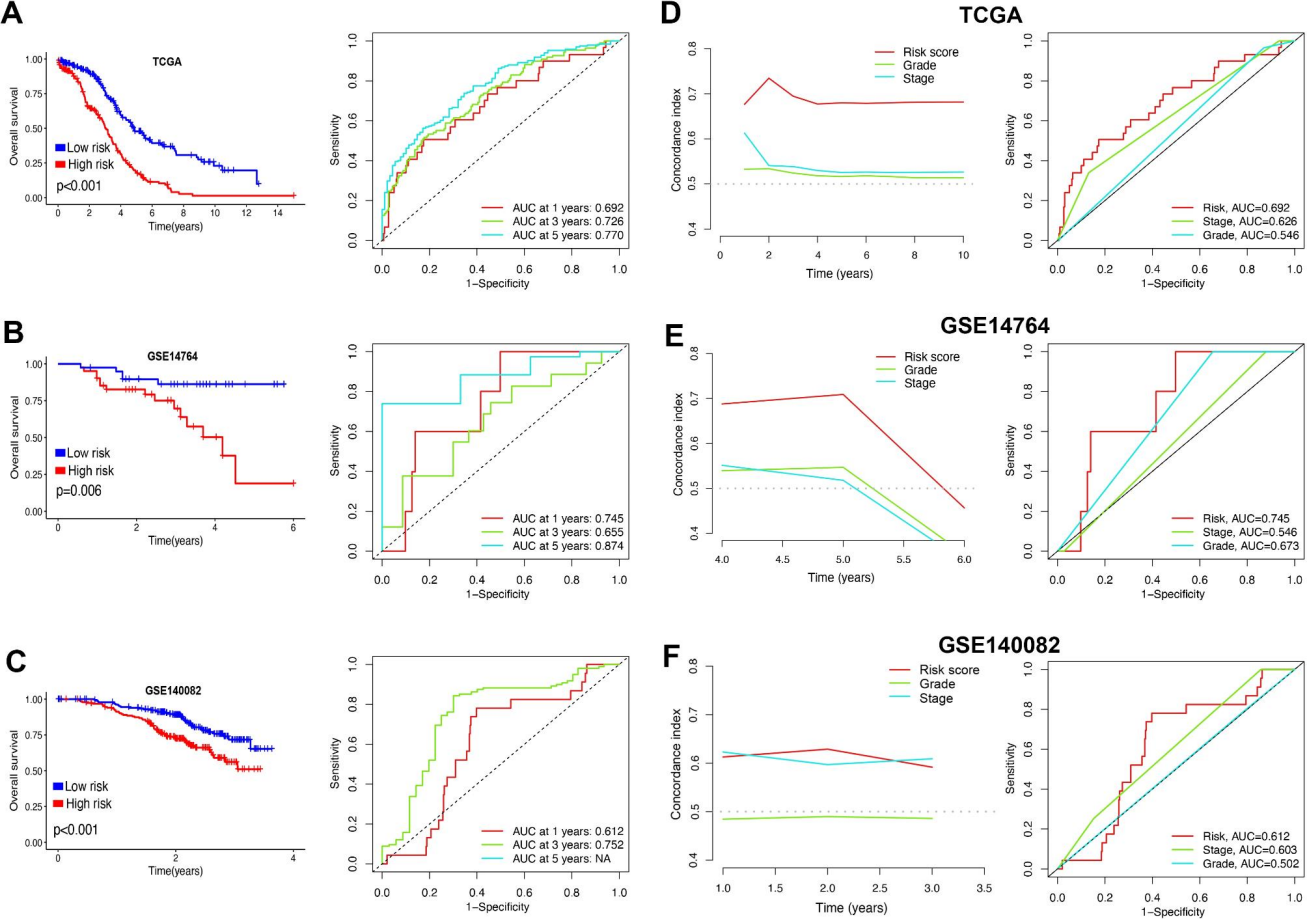
**Evaluation of the performance of macrophages-related signature (MRS).** The survival curve and corresponding TimeROC curve of ovarian cancer with high and low risk score in TCGA (**A**), GSE14764 (**B**) and GSE140082 (**C**) cohort. C-index and clinical ROC curve evaluated the discrimination of MRS in predicting the overall survival rate of ovarian cancer patients in TCGA (**D**), GSE14764 (**E**) and GSE140082 (**F**) cohort

**Fig. 5:**
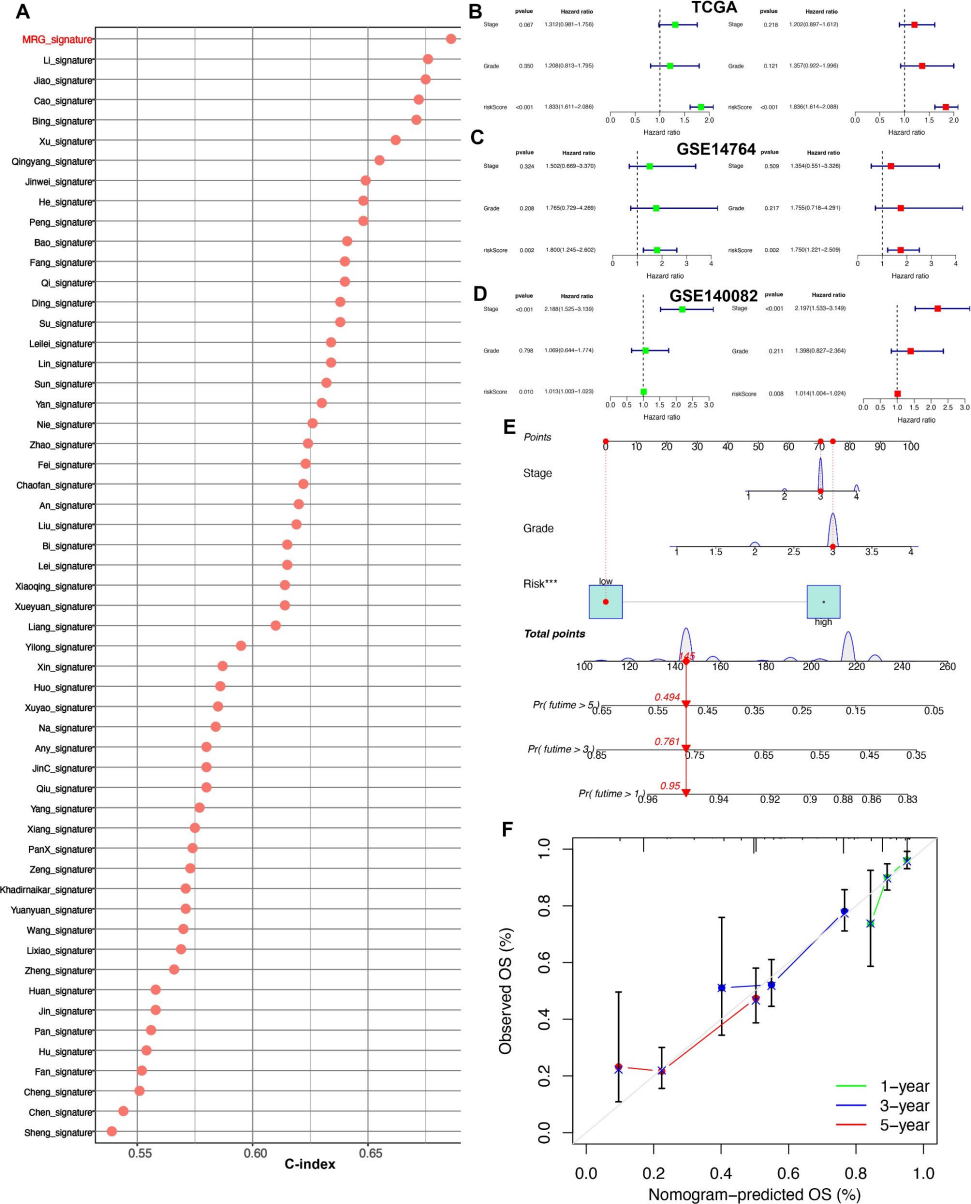
**Evaluation of the performance of macrophages-related signature (MRS).** (**A**) C-index of MRS and other established signatures evaluated the overall survival rate of ovarian cancer patients. Univariate and multivariate cox regression analysis considering grade, stage and MRS in TCGA (**B**), GSE14764 (**C**) and GSE140082 (**D**) cohort. (**E**-**F**) construction of survival prediction nomogram in ovarian cancer considering grade, stage and MRS for predicting the 1-, 3-, and 5-year OS rate

In TCGA cohort, ovarian cancer patients with high risk score had a poor OS rate with the AUCs of 1-, 3-, and 5-year ROC curve being 0.692, 0.726 and 0.774, respectively (Fig. 4A, p < 0.001). Moreover, high risk score indicated a poor OS rate in ovarian cancer patients based on GSE14674 cohort, with the AUCs of 1-, 3-, and 5-year ROC curve being 0.745, 0.655 and 0.874, respectively (Fig. 4B, p = 0.006). Similar results were obtained in GSE140082 dataset, and ovarian cancer patients with high risk score had a poor OS rate, with the AUCs of 1-, and 3-year ROC curve being 0.612 and 0.752, respectively (Fig. 4C, p < 0.001). Further analyses revealed that the C-index of risk score was higher than that of grade and stage in TCGA, GSE14764 and GSE140082 cohort (Fig. 4D F). Compared with grade and stage, the AUC value of risk score in clinical ROC curve was higher in TCGA, GSE14764 and GSE140082 cohort (Fig. 4D F). These evidences revealed that our MRS may have a better performance in predicting the OS rate of ovarian cancer compared with grade and stage. We then compared the C-index of our MRS and 55 prognostic signatures that have been established for ovarian cancer (Supplementary Table 3). As shown in Fig. 5A, the C-index of our MRS was higher than all of these prognostic signatures, which suggested the better performance of our MRS in predicting the clinical outcome of ovarian cancer than almost of other models. Moreover, univariate and multivariate cox regression analysis indicated that risk score could act as an independent risk factor for ovarian cancer in TCGA (Fig. 5B), GSE14764 (Fig. 5C) and GSE140082 (Fig. 5D) cohort. Using MRS, stage and grade, we then constructed a survival prediction nomogram (Fig. 5E), with which the clinicians may predict the mortality of ovarian cancer patients. Compared with the idea curve, our calibration curves had a relative well predictive value in the 1-, 3-, and 5-year OS rate (Fig. 5F).

### Dissection of MRS-based tumor microenvironment in ovarian cancer

**Fig. 6:**
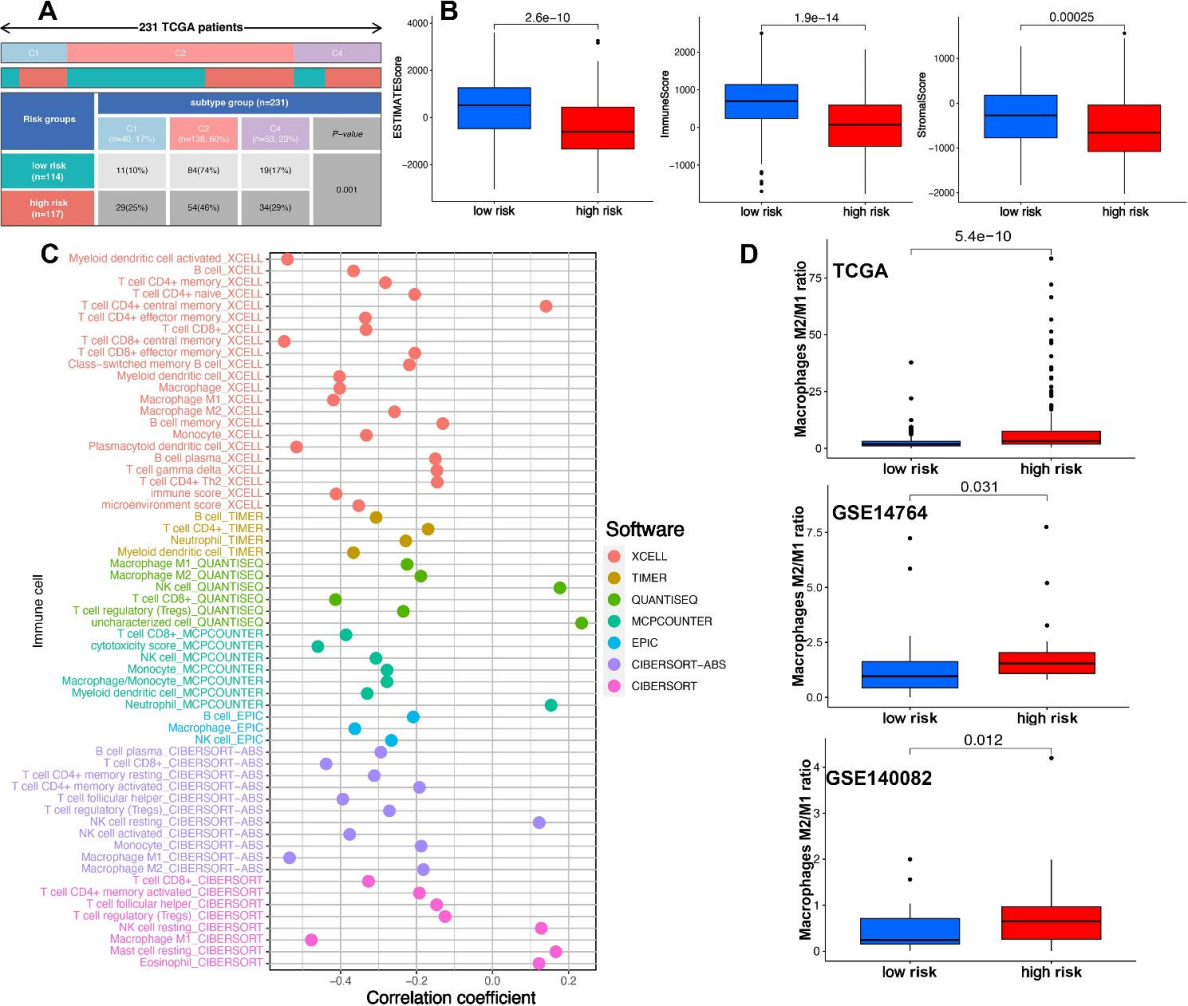
**The correlation between tumor microenvironment (TME) and macrophages-related signature (MRS).** (**A**) The difference of high and low risk score in tumor immune landscape. (**B**) The TME score in ovarian cancer patients with high and low risk score. (**C**) The correlation between MRS and the level of immune cells in ovarian cancer. (**D**) The level of macrophages M2/M1 proportion in ovarian cancer patients with high and low risk score in TCGA, GSE14764 and GSE140082 cohort

Tumor immune landscape could be divided into wound healing(C1), IFN-g dominant(C2), inflammatory(C3), lymphocyte depleted(C4), immunologically quiet(C5) and TGF-b dominant(C6) [27]. As shown in Fig. 6A, C2 ranked for the largest proportion in TCGA ovarian cancer cases with low risk score while C4 ranked a higher proportion in high risk score group (p = 0.001). Compared with ovarian cancer patients with high risk score, patients with low risk score had a higher ESTIMATEscore, immunescore, and stromalscore (Fig. 6B, all p < 0.05). The correlation between risk score and immune cell was shown in Fig. 6C, suggesting a negative correlation between risk score and the abundance of most immune cells. As macrophages M2/M1 proportion play a vital role in tumor progression, prognosis and immunotherapy [9–11]. We then compared the level of macrophages M2/M1 proportion in two group in ovarian cancer. As expected, ovarian cancer patients with high risk score had a higher level of macrophages M2/M1 proportion in TCGA, GSE14764 and GSE140082 cohort (Fig. 6D), suggested that ovarian cancer patients in high risk score had a higher possibility of tumor progression. All in all, low risk score may be a relatively “hot” tumor phenotype compared with high risk score in ovarian cancer.

### MRS-based treatment strategy for ovarian cancer

**Fig. 7:**
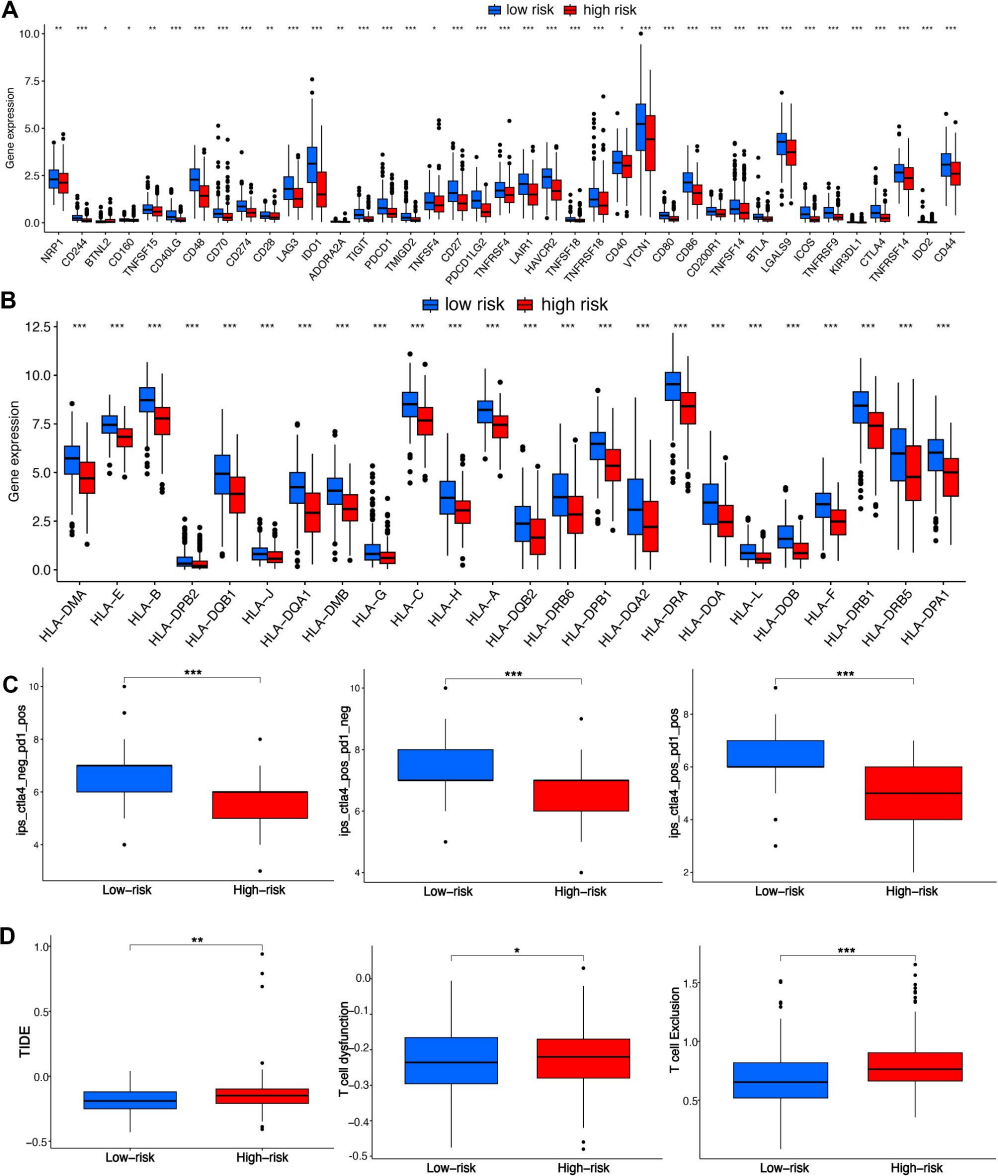
**Macrophages-related signature (MRS)-based treatment strategy for ovarian cancer.** The level of immune checkpoints (**A**), HLA-related genes (**B**), immunophenoscore (**C**) and TIDE score (**D**) in ovarian cancer patients with high and low risk score. *p < 0.05, **p < 0.01, ***p < 0.001

**Fig. 8:**
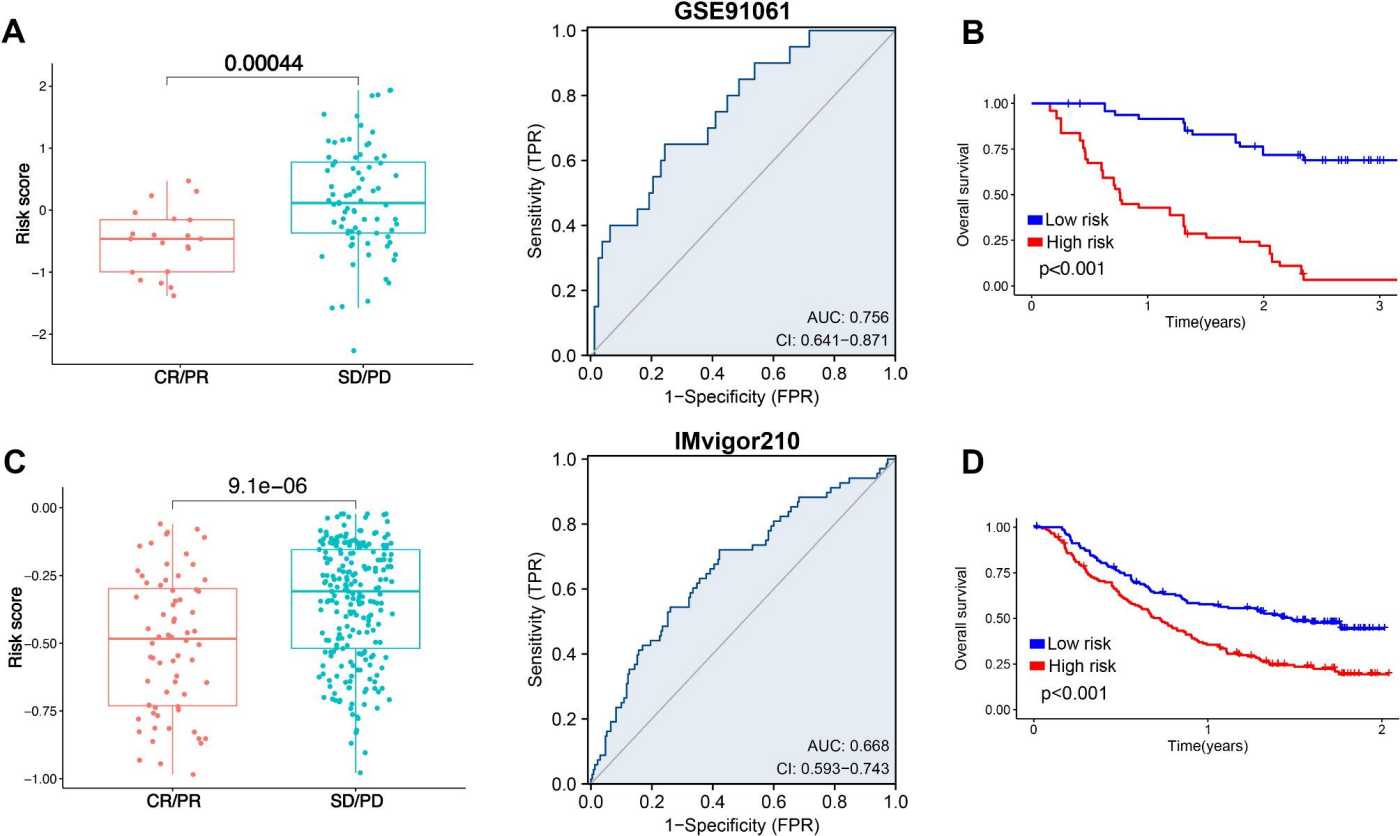
**Macrophages-related signature (MRS)-based treatment strategy for ovarian cancer.** Comparison of risk score in CR/PR and SD/PD group and corresponding ROC curve in GSE91061 (**A**) and IMvigor210 (**C**) cohort. The OS curve in patients with high and low risk score in GSE91061 (**B**) and IMvigor210 (**D**) cohort

**Fig. 9:**
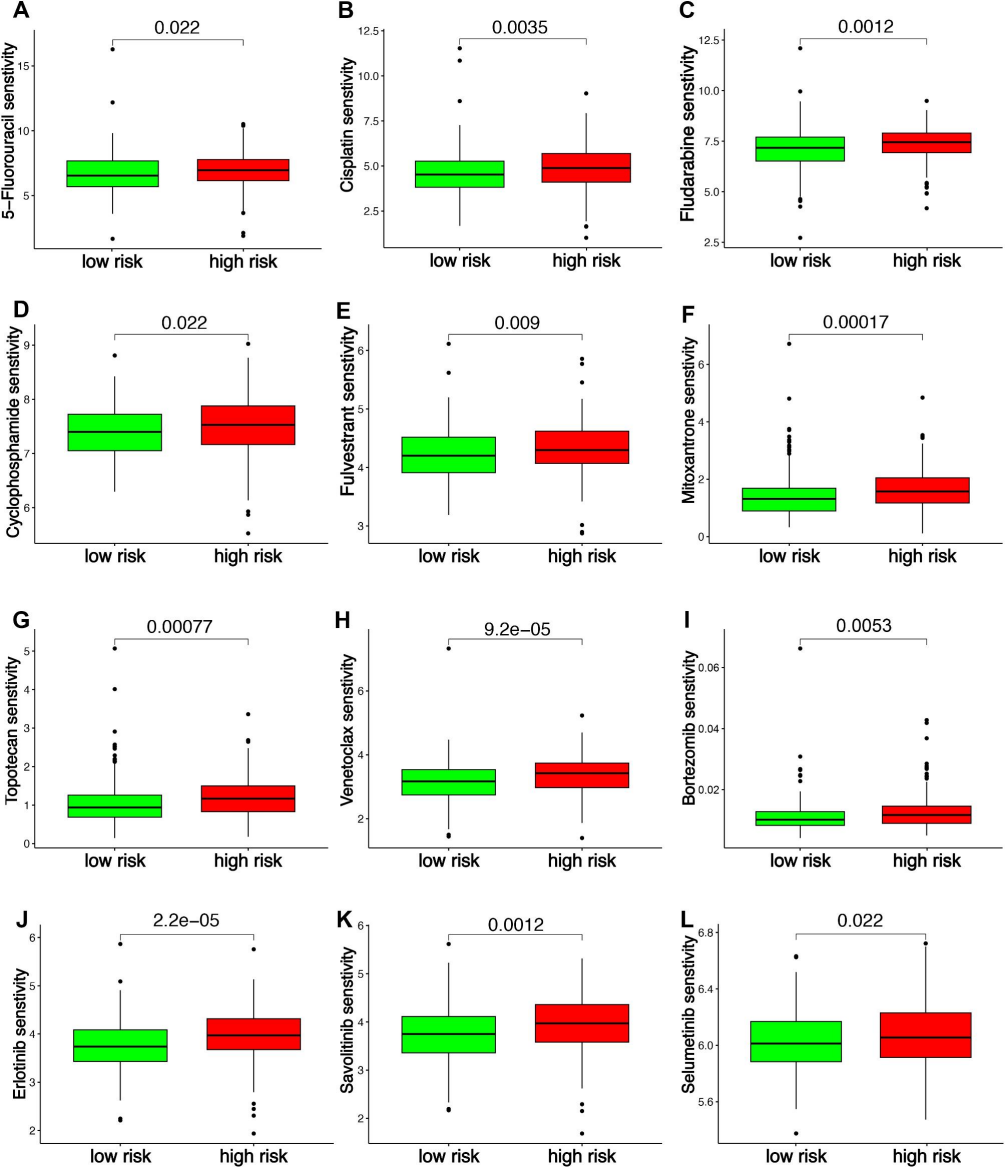
**Macrophages-related signature (MRS)-based treatment strategy for ovarian cancer.** The IC50 values of 5-Fluorouracil (**A**), Cisplatin(**B**), Fludarabine(**C**), Cyclophosphamide (**D**), Fulvestrant (**E**), Mitoxantrone (**F**), Topotecan(**G**), Venetoclax (**H**), Bortezomib (**I**), Erlotinib (**J**), Savolitinib (**K**), Selumetinib (**L**) in ovarian cancer patients with high and low risk score

Low risk score may be a relatively “hot” tumor phenotype compared with high risk score in ovarian cancer. In order to evaluate the difference of ovarian cancer with low and high risk score in immunotherapy, we used several indicators, including immune checkpoints, HLA-related genes, immunophenoscore (IPS), TIDE score, and IC50. Higher level of immune checkpoints, HLA-related genes and IPS and lower level of TIDE score and IC50 indicated a better drug sensitivity. As a result, ovarian cancer patients with low risk score had a higher level of immune checkpoints and HLA-related genes versus that with high risk score (Fig. 7A and B, p < 0.05). Moreover, low risk score was associated with higher IPS of anti-CTLA4, anti-PD1 and anti-CTLA4/PD1 in ovarian cancer patients (Fig. 7C, p < 0.05). Further analyses revealed that ovarian cancer patients with low risk score had a lower TIDE score and a lower score of T cell dysfunction and exclusion (Fig. 7D, p < 0.05). These evidences may suggest ovarian cancer patients with low risk score may have a better response to immunotherapy. In order to further evaluate the role of MRS in predicting the immunotherapy response of cancer, we then used two immune related cohorts (GSE91061 and IMvigor210). As expected, the risk score in CR/PR group was downregulated versus that in SD/PD group (Fig. 8A, p = 0.00044), with an AUC of 0.756 in GSE91061cohort. Further analysis revealed that cancer patients with high risk score had a poor OS rate (Fig. 8B, p < 0.001). In IMvigor210 cohort, the risk score in CR/PR group was lower versus that in SD/PD group (Fig. 8C, p = 9.1e-6), with an AUC of 0.668. Moreover, high risk score indicated a low OS rate (Fig. 8D, p < 0.001). Thus, our MRS may serve as an indicator for immunotherapy response. We then explored the IC50 values of the common drugs for ovarian cancer treatment. As a result, the IC50 values of 5-Fluorouracil, Cisplatin, Fludarabine, Cyclophosphamide, Fulvestrant, Mitoxantrone, Topotecan, Venetoclax, Bortezomib, Erlotinib, Savolitinib, Selumetinib were lower in ovarian cancer patients with low risk score compared with that with high risk score (Fig. 9A L, all p < 0.05), suggesting that ovarian cancer with low risk score may be more sensitive to chemotherapy, endocrinotherapy, target therapy.

### MRS-based mutation landscape for ovarian cancer

Supplementary Fig. 2A-2B showed the mutation landscape of ovarian cancer patients with low and high risk score. The three most frequently mutated genes were TP53, TTN, CSMD3. Compared with patients with high risk score, ovarian cancer patients with low risk score had a higher TMB score (Supplementary Fig. 2C, p < 0.047). TMB score was negatively correlated with risk score in ovarian cancer (Supplementary Fig. 2D, p = 0.0045). Moreover, ovarian cancer patients with low TMB score and high risk score had a poor OS rate (Supplementary Fig. 2E-2 F, all p < 0.05).

### The functional enrichment difference in low and high risk score in ovarian cancer

GSEA was performed to explore the difference of potential functional enrichment in low and high risk score group, which may clarify why low and high risk score group had a significant difference in clinical outcome, treatment response, and mutation landscape. As shown in Supplementary Fig. 3A, high risk score was mainly correlated with basal cell carcinoma, hedgehog signaling pathway, melanogenesis and ribosome. While low risk score was mainly correlated with immune related pathways, including antigen processing and presentation, and cytokine-cytokine receptor interaction (Supplementary Fig. 3B).

### MRS-based unsupervised clustering

Consensus clustering was performed to explore unidentified subtypes of ovarian cancer based on the expression pattern of 27 genes in MRS. Base on the consensus CDF and delta area, k = 2 was suggested as the optimal number to cluster ovarian cancer patients, suggesting that ovarian cancer the patients could be well classified into two clusters (Supplementary Fig. 4A-4B). Significant difference was found between OS rate and these two clusters (Supplementary Fig. 4C, p < 0.001). PCA analysis and tSNE analysis demonstrated significant differences of MRS gene profile between the two clusters (Supplementary Fig. 4D). As shown in Supplementary Fig. 4E-4G, ovarian cancer in Cluster 1 was associated with high level of most of immune cells, ESTIMATEscore, immunescore, and immune checkpoints (all p < 0.05).

### Validation of the expression and prognostic value of candidate markers

As shown in Supplementary Fig. 5A, the protein level of CITED2, LYVE1, OGN, RAP1A, and TBC1D22A were higher in ovarian cancer tissues than that in normal tissues. Further analysis showed that ovarian cancer patients with high level of CITED2, LYVE1, OGN, RAP1A, and TBC1D22A had a poor prognosis that that with level of CITED2, LYVE1, OGN, RAP1A, and TBC1D22A (Supplementary Fig. 5B, all p < 0.05).

## Discussion

Despite many approaches (surgery, chemotherapy, endocrinotherapy and immunotherapy) have been used to manage ovarian cancer patients, more than half of ovarian cancer patients will suffer from relapse and metastasis after standard of care therapies and the 5-year survival rate is only about 30% [4] The FIGO staging system. is a conventional approach for clinicians to make therapeutic and surveillance strategy. Heterogeneous clinical outcomes within the same stage of this approach may result in potential overtreatment or undertreatment. Until now, there are limited effective biomarker for predicting the prognosis and drug sensitivity of ovarian cancer clinically. As macrophages M2/M1 proportion plays a vital role in tumor progression, prognosis and immunotherapy [9–11], exploring the correlation between macrophages-related genes and the prognosis and therapy response in ovarian cancer may provide novel approaches for the management of ovarian cancer.

WGCNA analysis was performed to identify macrophage related genes. Based on these genes, univariate cox analysis was performed to identify potential prognostic biomarkers for ovarian cancer. After that, we developed an accurate and stable prognostic MRS. As a result, the model constructed by the combination of CoxBoost and StepCox[forward] was suggested as the optimal model with the highest average C-index of 0.63. MRS serve as an independent risk factor for the prognosis of ovarian cancer. Compared with stage and grade, the current MRS had a better performance in predicting the overall survival rate of ovarian cancer.

Actually, a lot of prognostic models had been developed for ovarian cancer. Xiang et al. developed a prognostic metabolism-related signature for serous ovarian cancer [28]. Another autophagy-related signature could serve as a prognostic biomarker for ovarian cancer [29]. Ferroptosis-related signature could predict the prognosis and immune microenvironment for ovarian cancer [30]. Moreover, glycometabolism-related signature [31], RNA binding protein-associated signature [32], and oxidative stress-related signature [33] could serve as a prognostic biomarker for ovarian cancer. Compared with other 55 random published signatures, the current MRS had a highest C-index, demonstrating the potential of MRS as promising surrogate for evaluating the prognosis of ovarian cancer patients clinically.

Immunotherapy was suggested as one of promising approaches for the treatment of cancer. Targeting immune checkpoint molecules could reinvigorate anti-tumor immunity and aid clearance of tumor [34]. Numerous inti-PD-L1/PD1 therapeutics, including nivolumab and pembrolizumab, have been approved for the first-line therapy of cancer [35, 36]. However, ovarian cancer response to immunotherapy is limited, needing further exploration. In order to evaluate the role of MRSs in predicting the immunotherapy of ovarian cancer, we used several indicators, including immune checkpoints, IPS, TIDE score and TMB score. IPS was a superior predictor of response to anti- CTLA-4 and anti-PD-1antibodies and high IPS indicated a better response to immunotherapy [37]. TIDE could predict the outcome of cancer patients treated with first-line anti-PD1 or anti-CTLA4 drugs [38, 39]. Low TIDE score indicated a better response to immunotherapy. higher TMB was associated with better overall survival across multiple cancer types [40]. In our study, low risk score indicated a higher TME score, higher level of immune cells, higher IPS, higher TMB score, and lower TIDE score. Moreover, in GSE91061 and IMvigor210 dataset, the risk score in CR/PR group was lower than that in SD/PD group. These evidences may suggest that ovarian cancer patients with low risk score may have a better response to immunotherapy.

As chemotherapy and endocrinotherapy were one of most vital therapeutic measures for ovarian cancer. We also analyzed the IC50 value of common drugs in high and low risk group of ovarian cancer. As a result, the IC50 values of 5-Fluorouracil, Cisplatin, Fludarabine, Cyclophosphamide, Fulvestrant, Mitoxantrone, Topotecan, Venetoclax, Bortezomib, Erlotinib, Savolitinib, Selumetinib were lower in ovarian cancer patients compared with that with high risk score, suggesting that ovarian cancer with low risk score may be more sensitive to chemotherapy, endocrinotherapy, and target therapy.

Some limitations could be found in our study. The patients were retrospectively recruited, which may inevitably lead to bias to some extent. It would be better to verify the prognostic MRS using clinical dataset.

## Conclusion

All in all, the current study constructed a powerful prognostic MRS for ovarian cancer using 10 machine learning algorithms. This MRS could predict the prognosis and drug sensitivity in ovarian cancer.

### Electronic supplementary material

Below is the link to the electronic supplementary material.

Supplementary Material 1

Supplementary Material 2

Supplementary Material 3

Supplementary Material 4

## Data Availability

The analyzed data sets generated during the study were sourced from the TCGA database (https://portal.gdc.cancer.gov/repository) and GEO database (https://www.ncbi.nlm.nih.gov/geo).
