# Supplementary material for: A macrophage related signature for predicting prognosis and drug sensitivity in ovarian cancer based on integrative machine learning
Source: BMC Med Genomics. 2023 Oct 2;16:230. doi: 10.1186/s12920-023-01671-z (PMC10544447; doi:10.1186/s12920-023-01671-z)
Supplement: Supplementary file 1 — Supplementary Material 1 [file 12920_2023_1671_MOESM1_ESM.docx]

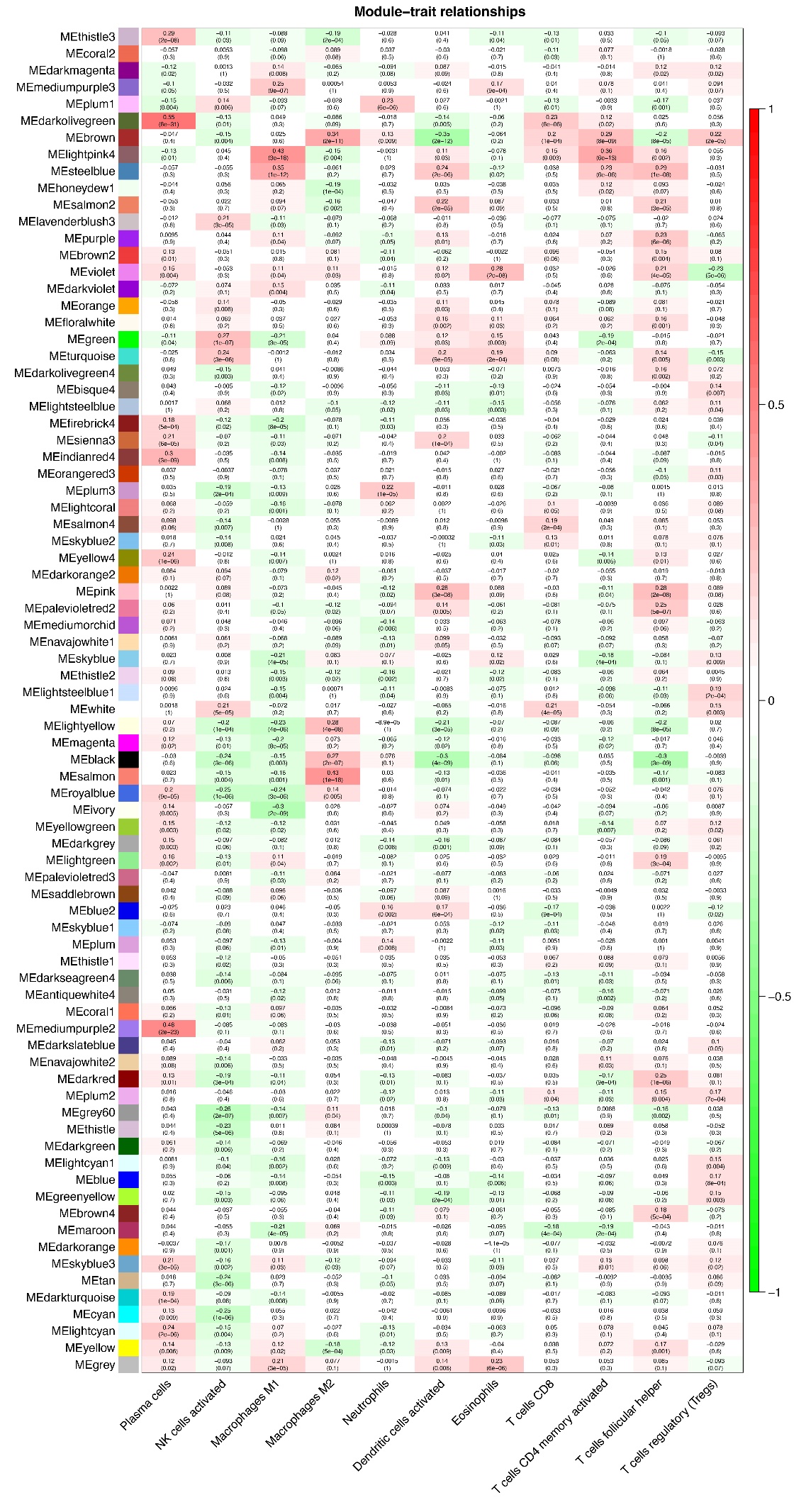


**Supplementary Figure 1. Heatmap showed the correlation between each module and immune cells.**


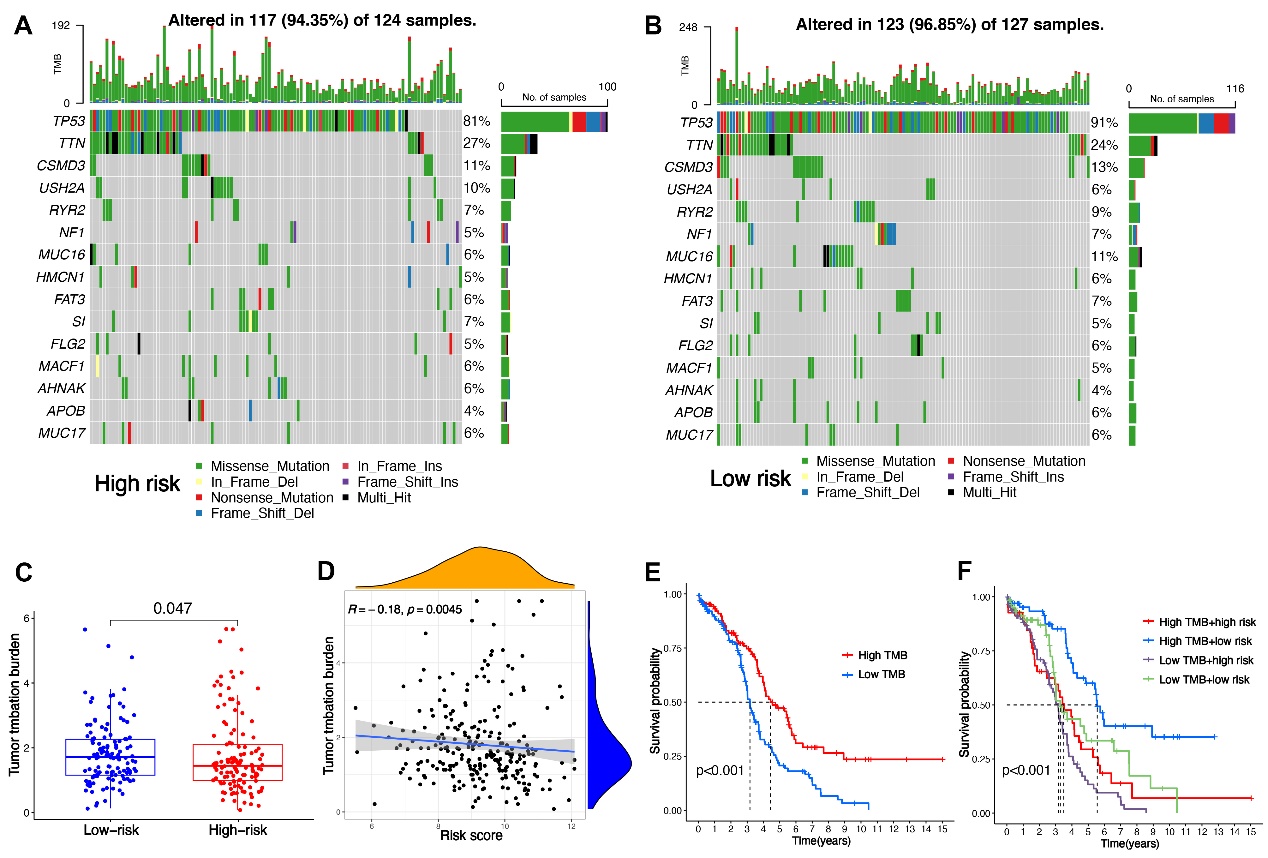


**Supplementary Figure 2. The correlation between genetic mutation and macrophages-related signature (MRS).** (A-B) Genetic landscape in ovarian cancer patients with high and low risk score. (C-D) The correlation between tumor mutational burden and risk score in ovarian cancer. (E-F) The survival curve in ovarian cancer patients with different tumor mutational burden and risk score.


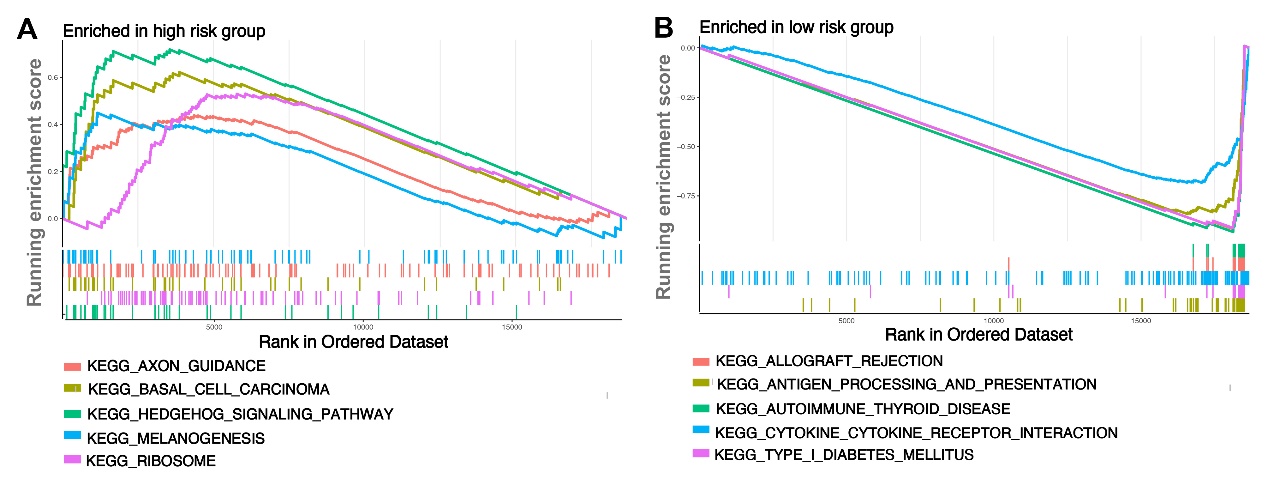


**Supplementary Figure 3. The enriched items in GSEA analysis.** The enriched items in the KEGG pathways in high (A) and low (B) risk score group.


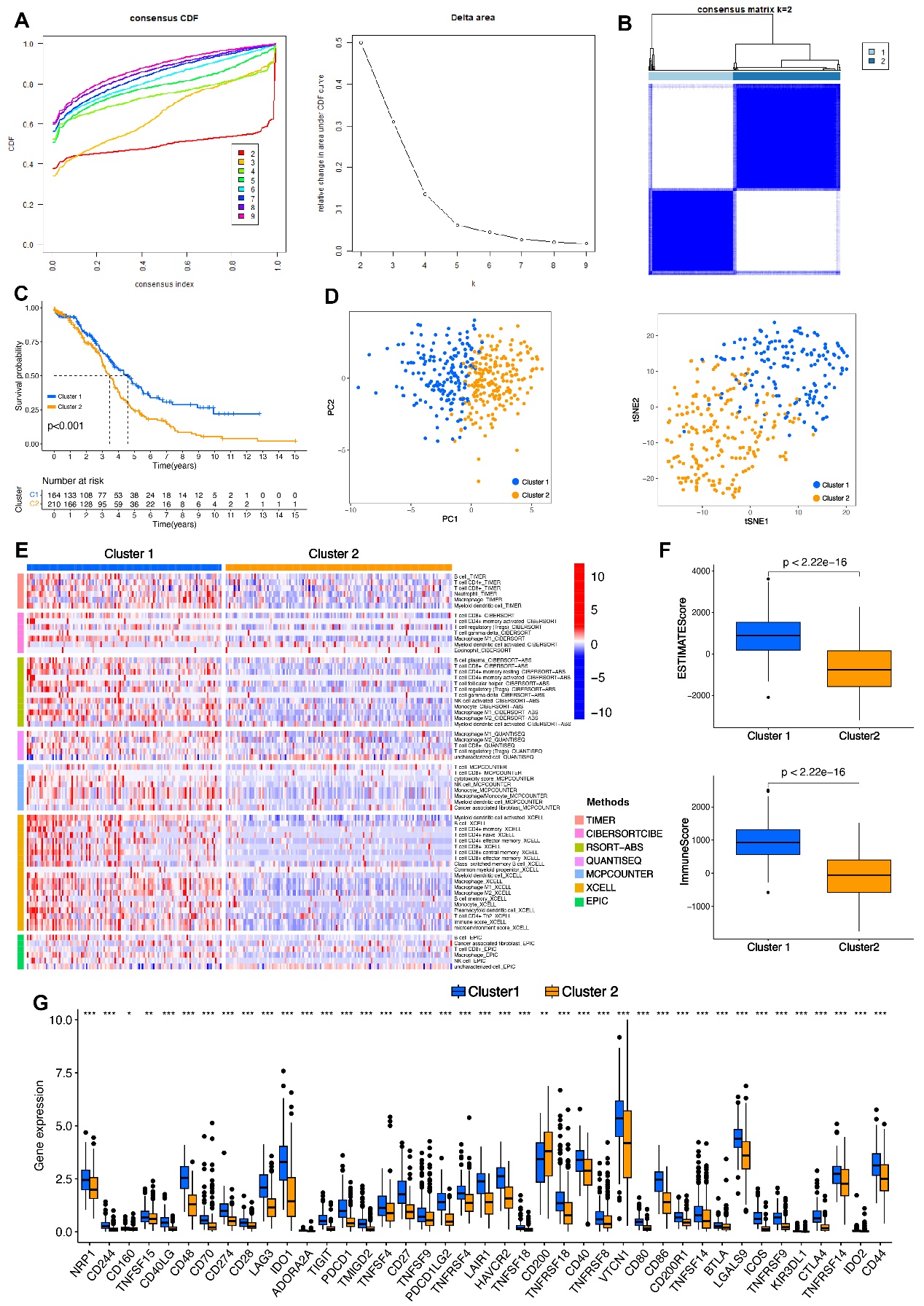


**Supplementary Figure 4. macrophages-related signature (MRS)-based unsupervised clustering.** (A-B) The consensus CDF, delta area and heatmap in unsupervised clustering analysis. (C) Survival curve in ovarian cancer patients in different clusters. (D) PCA analysis and tSNE analysis demonstrated significant differences of MRS gene profile between the two clusters. (E-G) The level of most of immune cells, ESTIMATEscore, immunescore, and immune checkpoints in ovarian cancer patients in different clusters.


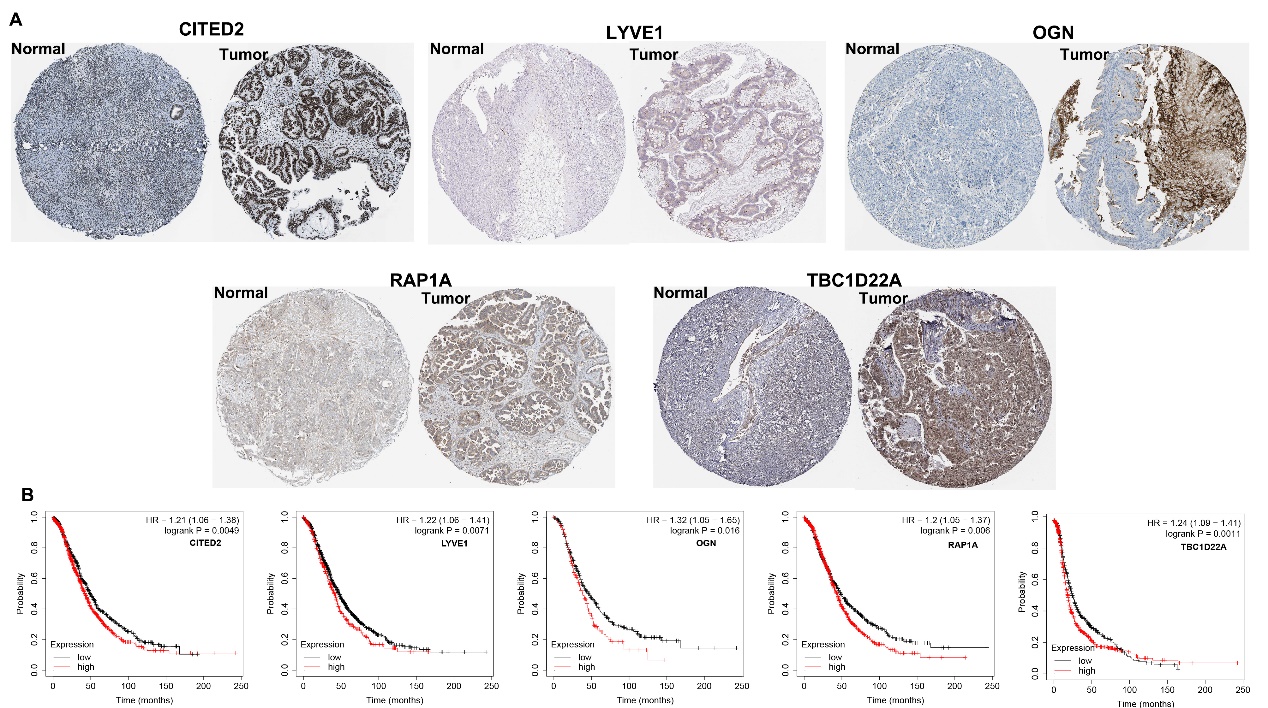


**Supplementary Figure 5. Validation of the expression and prognostic value of candidate markers.** (A) The immunohistochemistry of CITED2, LYVE1, OGN, RAP1A, and TBC1D22A in ovarian cancer tissues and normal tissues. (B) Ovarian cancer patients with high level of CITED2, LYVE1, OGN, RAP1A, and TBC1D22A had a poor prognosis that that with level of CITED2, LYVE1, OGN, RAP1A, and TBC1D22A.

**Supplementary Table 1. WGCNA identified macrophage related markers in ovarian cancer.**

**Supplementary Table 2. The candidate genes in prognostic macrophage-related signature and corresponding their coefficients.**

**Supplementary Table 3. Other models had been established for ovarian cancer.**
